# In Vitro Analysis of Pressure Resistance in the Paul Glaucoma Implant and Ahmed ClearPath 250 With and Without Polypropylene Thread Inside the Tube

**DOI:** 10.1167/tvst.14.1.2

**Published:** 2025-01-07

**Authors:** Andi Masdipa, Sachiko Kaidzu, Masaki Tanito

**Affiliations:** 1 Department of Ophthalmology, Shimane University Faculty of Medicine, Izumo, Japan

**Keywords:** glaucoma surgery, glaucoma drainage devices (GDDs), pressure resistance, polypropylene thread, hypotony prevention

## Abstract

**Purpose:**

Pressure resistance characteristics of the Paul glaucoma implant (PGI) and Ahmed ClearPath 250 (ACP), with and without the insertion of polypropylene thread in their tubes, were evaluated.

**Methods:**

The in vitro flow pressure was evaluated at varying flow rates, both with and without threads (6-0 for PGI and 4-0 or 3-0 for ACP). Cross-sectional areas of the tube lumen and thread were measured to calculate pressure resistance using the Hagen–Poiseuille equation.

**Results:**

For the PGI without the thread, the pressure remained relatively low and constant across all flow rates. In contrast, with the insertion of a 6-0 thread, there was a significant increase in pressure resistance, with the pressure increasing from 7.5 mm Hg at 1 µL/min to 43.8 mm Hg at 5 µL/min. For the ACP, the pressure resistance remained relatively constant across all flow rates without a thread and with either 4-0 or 3-0 threads. However, the pressure was higher with 3-0 threads compared with a 4-0 thread. The actual measured pressures agreed well with theoretical values in the no-thread conditions, but were consistently higher than the theoretical values in the threaded conditions.

**Conclusions:**

Inserting polypropylene threads into the tubes of nonvalved glaucoma drainage devices significantly affects pressure resistance with various degree.

**Translational Relevance:**

PGI with a 6-0 polypropylene thread may not require external tube ligation to prevent hypotony, whereas ACP with a 4-0 thread likely requires additional ligation. Using a 3-0 thread in ACP may enhance pressure resistance sufficiently to avoid tube ligation, but this requires careful clinical consideration.

## Introduction

Glaucoma treatment using glaucoma drainage implants is gaining popularity as the variety of these devices increases, encompassing both nonvalved and valved options.^1^ The primary function of glaucoma drainage implants is to lower elevated intraocular pressure (IOP) in glaucoma patients. However, it is crucial to avoid excessive reduction of IOP, which can lead to hypotony, a potentially harmful condition.^1^^,^^2^ Consequently, numerous clinical studies have been published on the outcomes of glaucoma drainage device (GDD) implantation.^3^^–^^7^ These studies provide valuable insights into the effectiveness and safety of GDDs. To optimize their use and achieve target IOP after implantation, a comprehensive understanding of the design, size, materials, and modifications of these devices is essential.^2^

The Molteno implant was the first GDD with a long tube and plate, followed by the Baerveldt glaucoma implant (BGI), both of which are nonvalved GDDs. The first valved GDD was the Krupin–Denver valve implant, succeeded by the Ahmed glaucoma valve (AGV).^1^ The BGI, a widely used nonvalved GDD, has an inner tube diameter of 0.32 mm, while the AGV, a popular valved GDD, has an inner tube diameter of 0.305 mm.^8^^,^^9^ Both devices are commonly used and frequently compared by glaucoma surgeons, having proven effectiveness in lowering IOP. However, regarding hypotony complications, the AGV has demonstrated more favorable outcomes compared with the BGI.^5^^,^^6^^,^^10^^,^^11^ In our previous study, we evaluated the pressure resistance characteristics of the AGV.^12^ Additionally, some studies have focused on assessing the pressure resistance of the BGI.^13^^,^^14^

Recently, new types of nonvalved GDDs have been developed, including the Paul glaucoma implant (PGI) and the Ahmed ClearPath 250 (ACP), which are designed to meet various clinical needs.^15^^–^^17^ Although both devices are made of silicon, the internal diameter of the ACP 250 tube (0.305 mm) is significantly larger than that of the PGI tube (0.127 mm).^17^^,^^18^ In clinical practice, polypropylene inserts are required for these two GDDs during implantation: size 4-0 for the ACP, which is provided by the manufacturer, and size 6-0 or 7-0 for the PGI, which is not included by the manufacturer. Although the ACP is prefitted with 4-0 polypropylene, tube ligation is still necessary during the implantation procedure. This study aims to explore and compare the pressure resistance of the PGI and ACP, with and without polypropylene inside the tube, in an in vitro experimental setting.

### Materials and Methods

### Materials

In this study, the pressure resistance of two types of new GDDs was measured. Four samples of the PGI from Advanced Ophthalmic Innovations (Singapore) and four samples of the ACP from New World Medical (Rancho Cucamonga, CA) were used.

### Experimental Settings

**Figure 1.**
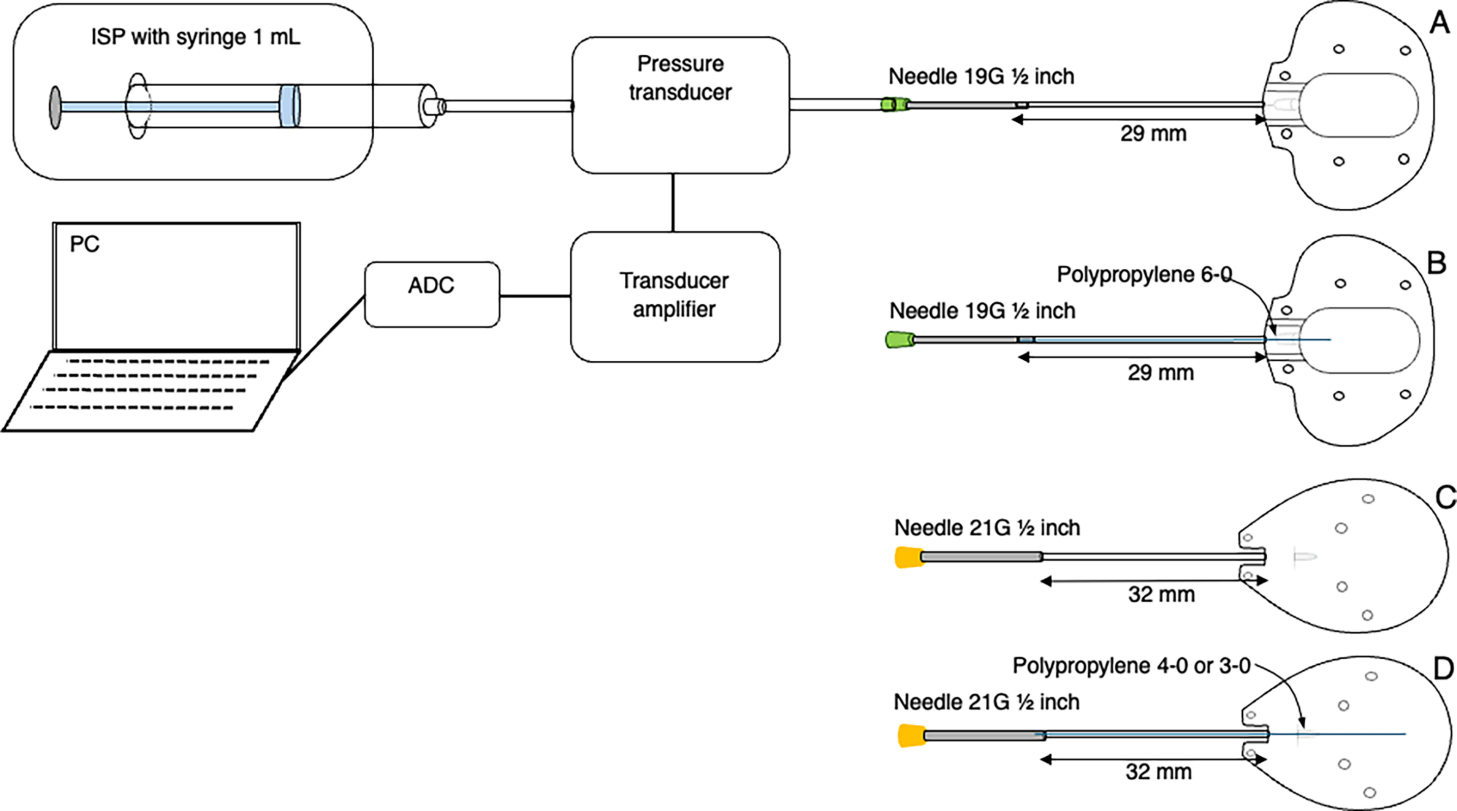
Experimental setup for pressure resistance measurement. (**A**) Paul glaucoma implant (PGI) without polypropylene thread. (**B**) PGI with 6-0 polypropylene thread. (**C**) ACP without polypropylene thread. (**D**) ACP with either 4-0 or 3-0 polypropylene thread. ADC, analog-to-digital converter; ISP, infusion syringe pump system; PC, personal computer.

The experiments were conducted in vitro using the same setup as described in our previous study.^12^^,^^19^^,^^20^ As shown in Figure 1, the experimental setup began with the infusion of physiological saline from 1-mL syringes (Terumo Corporation, Tokyo, Japan) connected to a syringe pump (SP101i, KD Scientific, Holliston, MA). The pressure measurement equipment consisted of a BLPR2 pressure transducer, a 4-channel amplifier (SYS TBM4M), an analog-to-digital converter (LAB-TRAX-4/16), and LabScribe2 software for pressure curve analysis, all supplied by World Precision Instruments, Sarasota, FL. Infusion tubes from JMS Co., Ltd. (Tokyo, Japan), were used to connect the 1-mL syringes to the pressure transducer and from the transducer to the GDD using appropriately sized needles. A 21G 1.5-inch needle (Figs. 1A and 1B) was used to connect the PGI, and a 19G 1.5-inch needle (Figs. 1C and 1D) was used to connect the ACP. Both types of needles were sourced from Terumo Corporation. These needles were selected to ensure that their inner diameter was slightly larger than the outer diameter of each GDD. To prevent leakage at the connection between the needle and the GDD, glue from TOMBOW PENCIL CO., Ltd. (Aichi, Japan) was applied.

The measurement procedure began with the infusion of 0.9% physiological saline into the experimental setup as shown in Figure 1, starting from the 1-mL syringe on the infusion syringe pump to the needle tip where the GDD tube tip was inserted. Before inserting the GDD tube tip, the amplifier transducer was calibrated to ensure a zero reading on the monitor. Each GDD was then subjected to physiological saline flow at rates of 1.0, 1.5, 2.0, 2.5, 3.0, 4.0, and 5.0 µL/min, both without and with a polypropylene thread inside the GDD tube. For the PGI tubes, 6-0 polypropylene (6-0 PROLEN, J&J MedTech, Tokyo, Japan) was inserted. The ACP 250 comes with a 4-0 polypropylene thread provided by the manufacturer, but a 3-0 polypropylene thread (3-0 PROLEN, J&J MedTech) was also used in the ACP 250.

### Cross-Sectional Area Measurement

**Figure 2.**
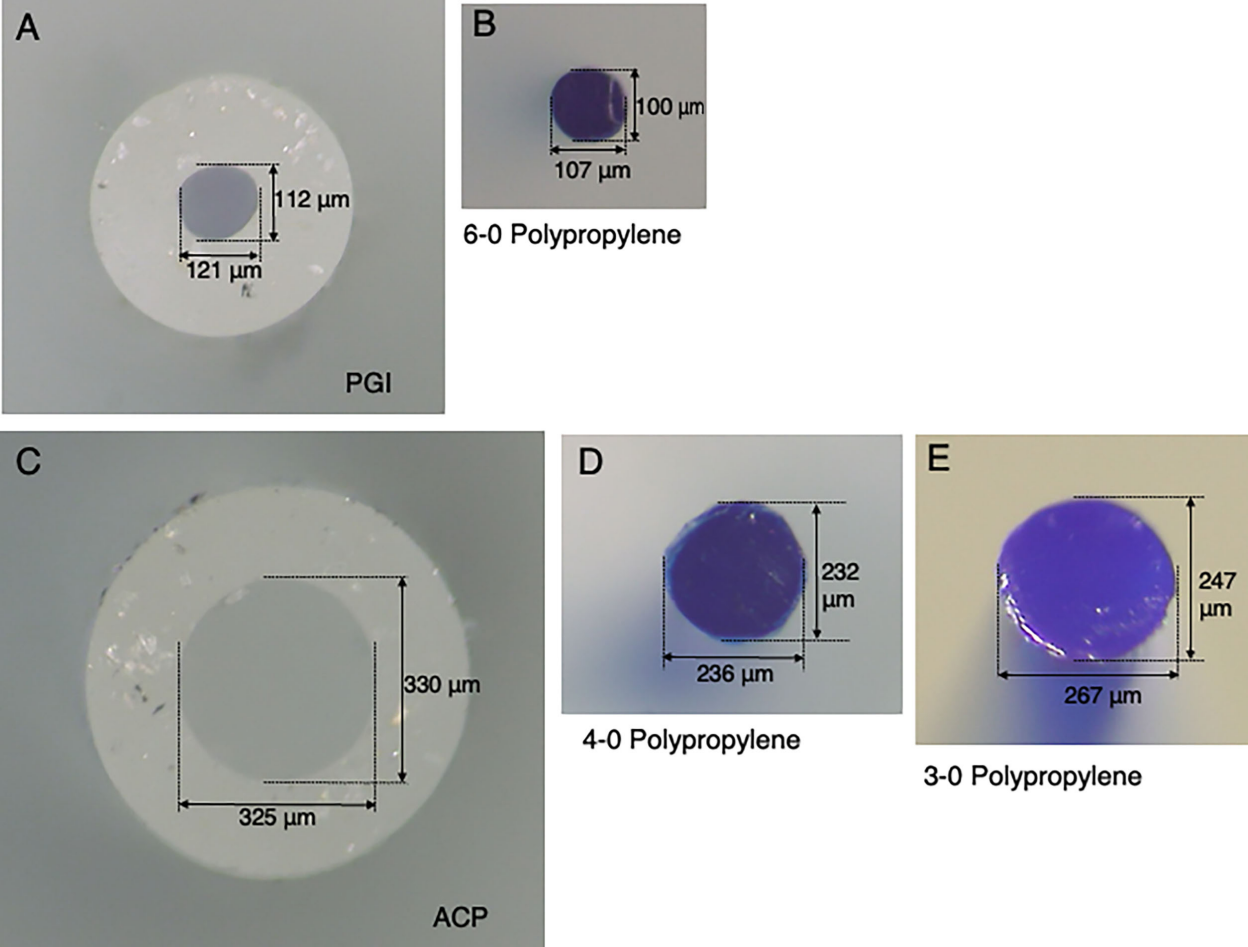
Representative microscopic images of tube or thread cross-sections. (**A**) Paul glaucoma implant (PGI). (**B**) The 6-0 polypropylene thread. (**C**) ACP. (**D**) The 4-0 polypropylene thread. (**E**) The 3-0 polypropylene thread.

Each sample of the PGI, ACP, and polypropylene thread underwent diameter measurements of the tube lumen or thread cross-section (Fig. 2). Measurements were performed using a multi-angle stereo microscope and digital camera system (VB-7010/VB-G25, Keyence Co. Ltd., Osaka, Japan) with 175× magnification. Owing to the elliptical appearance of the lumen of the GDD tube and the polypropylene thread under the microscope, both the transverse and horizontal diameters were measured (Fig. 2). The cross-sectional area (*A*) was calculated using the following equation:

(1) $$\begin{array}{c} A=\frac{1}{4}\pi \times H\times V, \end{array}$$

where *A* is the area, *H* is the horizontal diameter, and *V* is the vertical diameter.

### Statistical Analysis

The data were analyzed using JMP Pro 17.2 statistical software (JMP Statistical Discovery, Cary, NC). The results were presented as mean ± standard deviation, and comparisons were made using paired and unpaired *t* tests.

## Results

Table 1 presents the results of the pressure resistance measurements. For the PGI, flow pressure was measured without [PGI (−)] and with [PGI (+)] a 6-0 polypropylene thread inside the tube. The flow pressure of PGI (−) remained relatively constant as the flow rate increased from 1 µL/min to 5 µL/min. In contrast, the pressure of PGI (+) showed a significant increase, rising from 7.5 mm Hg at 1 µL/min to 43.8 mm Hg at 5 µL/min. For the ACP, flow pressure was measured without [ACP (−)] and with either a 4-0 [ACP (+4)] or 3-0 [ACP (+3)] polypropylene thread inside the tube. Unlike the PGI (+) condition, in all three ACP conditions, the flow pressures remained relatively constant as the flow rate increased from 1 µL/min to 5 µL/min. The pressure was higher in both ACP (+4) and ACP (+3) compared with ACP (−), and ACP (+3) consistently showed higher pressures than ACP (+4) across all flow rates. Except at lower flow rates, the pressures were higher in PGI (−) than in ACP (−). Across all flow rates tested, pressures were higher in PGI (+) than in ACP (+4) and ACP (+3).

**Table 1.** Comparison of Measured Tube Pressures Under Various Conditions

	Pressure		*P* Value*	Pressure			*P* Value*			*P* Value (PGI vs. ACP) **		
Flow, µL/Min	PGI (−)	PGI (+)	PGI (−) vs. (+)	ACP (−)	ACP (+4)	ACP (+3)	ACP (−) vs. (+4)	ACP (−) vs. (+3)	ACP (+4) v.s (+3)	PGI (−) vs. ACP (−)	PGI (+) vs. ACP (+4)	PGI (+) vs. ACP (+3)
1.0	0.7 ± 0.4	7.5 ± 0.5	0.0004	0.6 ± 0.2	2.2 ± 0.8	3.3 ± 0.7	0.02	0.003	0.03	0.9	<0.0001	0.003
1.5	0.8 ± 0.4	11.2 ± 0.9	0.0002	0.8 ± 0.3	2.4 ± 0.8	3.9 ± 0.5	0.008	0.0006	0.02	0.9	<0.0001	0.003
2.0	1.0 ± 0.4	17.1 ± 2.7	0.002	0.9 ± 0.3	2.5 ± 0.8	4.3 ± 0.4	0.006	0.0006	0.02	0.8	0.001	0.004
2.5	1.3 ± 0.5	21.0 ± 4.0	0.003	1.0 ± 0.3	2.6 ± 0.8	4.6 ± 0.5	0.01	0.0008	0.03	0.4	0.002	0.004
3.0	1.6 ± 0.5	26.0 ± 4.9	0.002	1.0 ± 0.3	2.8 ± 1.0	5.2 ± 0.7	0.02	0.002	0.04	0.1	0.002	0.003
4.0	1.9 ± 0.6	33.8 ± 6.4	0.002	1.1 ± 0.3	3.0 ± 1.1	5.9 ± 0.9	0.01	0.003	0.04	0.047	0.002	0.003
5.0	2.3 ± 0.5	43.8 ± 8.7	0.003	1.1 ± 0.3	3.1 ± 1.1	6.4 ± 0.8	0.02	0.002	0.03	0.01	0.002	0.003

Pressure values are expressed in mean ± standard deviation (mm Hg).

* *P* value obtained by paired *t* test.

** *P* value obtained by unpaired *t* test.

Table 2 displays the remaining lumen area of the tube (ΔA), calculated based on the measured tube lumen area (A) and the cross-sectional area of the threads (A′) for both PGI and ACP. According to the measurements, the lumen area (A) of PGI (−) was eight times smaller than that of ACP (−). With the insertion of the polypropylene thread, the remaining lumen area (ΔA) of PGI (+) became 20 times smaller than that of ACP (+4) and 15 times smaller than ACP (+3). Because the value of A′ remained nearly constant across samples, a single A′ value was used for calculation in each condition.

**Table 2.** Remaining Lumen Area of the Tube (ΔA) Calculated Based on the Measured Tube Lumen Area (A) and the Cross-Sectional Area of Threads (A′).

GDD	Lumen (A)	Thread (A')	ΔA = A − A'
PGI + thread 6-0	1.0 ± 0.06	0.8	0.2 ± 0.06
ACP + thread 4-0	8.3 ± 0.12	4.3	4.0 ± 0.12
ACP + thread 3-0	8.3 ± 0.12	5.2	3.1 ± 0.12

Data are expressed in mean ± standard deviation (10^−8^m^2^).

ACP, Ahmed ClearPath; GDD, glaucoma drainage implant; PGI, Paul glaucoma implant.

Table 3 provides the mathematical calculations of pressure resistance based on the Hagen–Poiseuille equation for the PGI and ACP tubes, both with and without the polypropylene thread inside the tube. For these calculations, the values of A and ΔA shown in Table 2 were used. In the PGI (−) condition, there was a slight but clear increase in pressure with increasing flow rates. In the PGI (+) condition, this relationship was more pronounced. However, no increase in pressure was observed in any of the three conditions for ACP. The actual measured pressures (Table 1) and the theoretically calculated results (Table 3) showed good agreement for the conditions without threads [i.e., PGI (−) and ACP (−)]. However, for the conditions with threads [i.e., PGI (+), ACP (+4), and ACP (+3)], the actual measured pressures were consistently higher than the theoretical values.

**Table 3.** Theoretically Estimated Tube Pressures Using the Hagen-Poiseuille Equation

Flow, µL/Min	PGI (−)	PGI (+)	ACP (−)	ACP (+4)	ACP (+3)
1.0	0.9 ± 0.1	5.8 ± 3.2	0.02 ± 0.000	0.19 ± 0.02	0.44 ± 0.05
1.5	1.3 ± 0.2	8.7 ± 4.8	0.02 ± 0.001	0.29 ± 0.02	0.66 ± 0.07
2.0	1.7 ± 0.2	11.6 ± 6.4	0.03 ± 0.001	0.38 ± 0.03	0.88 ± 0.10
2.5	2.1 ± 0.3	14.5 ± 8.0	0.04 ± 0.001	0.48 ± 0.04	1.10 ± 0.12
3.0	2.6 ± 0.3	17.4 ± 9.6	0.04 ± 0.001	0.57 ± 0.05	1.31 ± 0.14
4.0	3.4 ± 0.4	23.2 ± 12.9	0.06 ± 0.002	0.76 ± 0.06	1.75 ± 0.19
5.0	4.3 ± 0.6	29.0 ± 16.1	0.07 ± 0.002	0.95 ± 0.08	2.19 ± 0.24

Data are expressed in mean ± standard deviation (mm Hg).

## Discussion

This study measured the pressure resistance of the PGI and ACP with and without polypropylene thread inside their tubes. As expected, the results demonstrated that the presence of polypropylene inserts significantly increased the pressure resistance for both PGI and ACP. Furthermore, when polypropylene was present inside the tube, the pressure resistance of the PGI was significantly higher than that of the ACP. This finding suggests that the characteristics of hypotony, which may occur after surgery with these GDDs, differ between the two implants.

In this study, pressure resistance refers to the pressure generated by the GDD in relation to the internal diameter and length of the tube and the fluid flow rate. For a straight cylindrical tube with laminar flow (i.e., no turbulence), dynamic pressure resistance can be calculated using the Hagen-Poiseuille equation, as follows:

(2) $$\begin{array}{c} \Delta P=P1-P2, \end{array}$$

(3) $$\begin{array}{c} \Delta P=\frac{8\mu LQ}{\pi r^{4}} \end{array}$$

(4) $$\begin{array}{c} A=\pi r^{2} \end{array}$$

(5) $$\begin{array}{c} \Delta P=\frac{8\pi \mu LQ}{A^{2}}. \end{array}$$

In (Equation 2), Δ*P* represents the pressure difference between *P1*, which is the IOP measured by the pressure transducer, and *P2*, which is the pressure at the distal end of the tube. (Equation 3) represents the Hagen–Poiseuille equation, where Δ*P* is the pressure resistance, *µ* is the fluid viscosity, *L* is the length of the tube, *Q* is the fluid flow rate, and *r* is the inner radius of the tube. However, in actual GDD samples, the tube diameter is not a perfect cylinder but rather elliptical. Therefore, by replacing the radius (*r*) with the cross-sectional area (*A*) in (Equation 4), the modified Hagen–Poiseuille equation used to calculate pressure resistance becomes (Equation 5). This equation is specifically applicable to laminar flow in tubes with small diameters, where the fundamental assumptions of the equation are well satisfied when the Reynolds number (*Re*) is less than 2000. The Reynolds number can be calculated using the following formula:

(6) $$\begin{array}{c} Re=\frac{\rho uD}{\mu }, \end{array}$$

where *ρ* is the fluid density, *u* is the average fluid velocity, *D* is the tube diameter, and *µ* is the dynamic viscosity of the fluid. In such cases, the Hagen–Poiseuille equation accurately describes the pressure resistance in the tube. For conditions without a thread, the pressure values derived from the equation (Table 3) closely matched the actual measured values for both PGI (−) and ACP (−) (Table 1). This confirms that the flow within the tube is laminar and unobstructed, demonstrating the equation's accuracy in predicting pressure resistance in nonvalved tubes. Accordingly, the main physical factors affecting this resistance are the tube's diameter and length.

In contrast, the values in Tables 1 and 3 do not align, particularly under the threaded (+) condition, with discrepancies between experimental and theoretical calculations summarized in Table 4. These differences make it difficult to accurately predict the flow pressure theoretically. The increase in pressure resistance inside the tube containing the threads is due to the change in flow shape from cylindrical to annular flow. This transformation also results in a reduction in the cross-sectional area of the flow (Table 2). Although the presence of the threads and the reduction in tube diameter prevent the flow from becoming fully laminar, mathematical calculations with an Re of less than 2000 indicate that the flow is not turbulent (Re > 4000). Therefore, the pressure resistance values in Table 3 for the GDD with thread (+) conditions did not use the conventional Hagen–Poiseuille equation for cylindrical tubes, but used the equation for pressure resistance in laminar flow in annular tubes, as shown below:

(7) $$\begin{array}{c} \Delta P=\;\frac{8\mu LQ}{\pi {\left(R_{o}^{2}-R_{i}^{2}\right)}^{2}}\times \frac{1}{ln\frac{R_{o}}{R_{i}}}. \end{array}$$

In (Equation 7), several parameters have been explained in Equation (3), where, *R_o_*​ represents the internal radius of the tube, and *R_i_* refers to the radius of the thread inside the tube. However, because both the tube and the thread are not perfectly circular but elliptical in shape, the area is calculated using the formula in (Equation 1). Therefore, (Equation 7) can be expressed as follows:

(8) $$\begin{array}{c} \Delta P=\;\frac{8\mu LQ}{\pi {\left(\frac{A}{\pi }-\frac{A^{'}}{\pi }\right)}^{2}}\times \frac{1}{ln\sqrt{\frac{A}{A^{'}}}}. \end{array}$$

In (Equation 8), *A* represents the cross-sectional area of the tube, and *A*′ is the cross-sectional area of the thread inside the tube. Another factor that leads to a lower theoretical calculation is that the position of the thread along the length of the tube cannot be fixed, whereas (Equation 8) assumes a model where the tube has a constant ring-shaped cross-section along its length. Our measurements indicate that the properties of the stent thread inserted into the tube are a critical determinant of flow pressure, providing insights for future device development or modifications. For example, flow pressure could vary by bending the thread, even with the same thread thickness. Additionally, creating fin-like slits on the thread surface may provide a unidirectional valve effect.

**Table 4.** Difference in Pressure Values Between Experimental and Theoretical Results

Flow, µL/Min	PGI (−)	PGI (+)	ACP (−)	ACP (+4)	ACP (+3)
1.0	0.2	1.7	0.6	2.0	2.9
1.5	0.1	2.5	0.8	2.2	3.3
2.0	0.0	5.5	0.8	2.1	3.4
2.5	0.0	6.5	0.9	2.1	3.5
3.0	0.1	8.6	0.9	2.3	3.9
4.0	0.0	10.6	1.0	2.2	4.1
5.0	−0.2	14.8	1.0	2.1	4.2

Experimentally measured values minus theoretically estimated values are expressed (mm Hg).

The lower pressure resistance observed in PGI (−) and ACP (−) suggests a risk of hypotony if these devices are used without any preventive measures. Clinical studies have shown that while PGI without polypropylene may achieve optimal IOP lowering, there remains a concern regarding the risk of hypotony.^21^^–^^23^ The insertion of a 6-0 polypropylene thread into the PGI tube has been found effective in preventing hypotony, without the need for additional measures such as tube ligation.^9^^,^^21^^–^^23^ This finding is well-supported by the flow pressure measurements of PGI (+) shown in Table 1.

For the ACP, the manufacturer provides a 4-0 polypropylene thread for stenting the tube. However, the pressure resistance of ACP (+4) remains relatively low (Table 1), which suggests a risk of postoperative hypotony. Clinically, external tube ligation is typically performed when implanting the ACP to enhance pressure resistance, even when a polypropylene thread is present inside the tube.^24^^,^^25^ Our measurements support this clinical practice, indicating that tube ligation is necessary for this device. In this study, the pressure resistance of the ACP was also measured with a 3-0 polypropylene thread, which is 20% thicker than the 4-0 thread, and a significant increase in flow pressure was observed. The use of the 3-0 thread is based on practices with the BGI, which also clinically employs a 3-0 thread with an internal diameter of 0.32 mm, compared with the ACP's internal diameter of 0.305 mm.^9^ Even with the use of a 3-0 polypropylene thread, the BGI still requires external tube ligation in clinical practice. Given that the ACP has a smaller internal tube diameter than the BGI, it may be possible to implant the ACP without tube ligation if a 3-0 thread is used as the tube stent. However, this approach would still require careful clinical judgment.

A limitation of this study is that it was conducted in vitro, and the experimental conditions differ from those in a clinical setting. When implanting a GDD, the tube is trimmed before implantation, which results in a lower pressure resistance in clinical practice compared with the values reported in this study. Additionally, in a clinical setting, the distal end of the tube and plate will be covered by conjunctival and Tenon's capsule tissues, whereas, in our experimental setup, the tube end was exposed to air. Furthermore, in a living eye, the production of aqueous humor can vary between cases, and factors such as tissue scarring and changes in the properties of aqueous humor also play a role. Therefore, the pressure resistance measured in this experiment does not directly indicate the IOP in a clinical situation.

For newly developed nonvalved GDDs, the pressure resistance measured in vitro aligns well with the predictions of the Hagen–Poiseuille equation when no thread is inserted inside the tube. However, when a polypropylene thread is inserted, pressure resistance increases, which does not correspond to the Hagen–Poiseuille equation due to more complex fluid dynamic factors. To prevent postoperative hypotony, PGI may not require external tube ligation when a 6-0 polypropylene thread is used as a stent. In contrast, ACP with a 4-0 polypropylene thread likely requires external ligation due to lower pressure resistance. The use of a 3-0 polypropylene thread instead of a 4-0 thread for ACP may potentially eliminate the need for tube ligation.
